# Application of Ethnobotanical Indices on the Use of Traditional Medicines against Common Diseases

**DOI:** 10.1155/2014/635371

**Published:** 2014-05-20

**Authors:** Imran Khan, Naser M. AbdElsalam, Hassan Fouad, Akash Tariq, Riaz Ullah, Muhammad Adnan

**Affiliations:** ^1^Department of Botany, Kohat University of Science and Technology, Kohat 26000, Pakistan; ^2^Riyadh Community College, King Saud University, Riyadh 11437, Saudi Arabia; ^3^Department of Chemistry, Biomedical Engineering Department, Faculty of Engineering, Helwan University, P.O. Box 11792, Helwan, Egypt; ^4^Department of Chemistry, Government College Ara Khel, FR Kohat, Khyber Pakhtunkhwa 26000, Pakistan

## Abstract

The present study was aimed at documenting the detailed ethnomedicinal knowledge of an unexplored area of Pakistan. Semistructured interviews were taken with 55 informants randomly chosen regarding detailed ethnomedicinal and sociocultural information. The study exposed 67 medicinal plant species used to prepare 110 recipes and the major modes of herbal formulation were decoction and powdering (20% each). The disease categories with the highest Fic values were gastrointestinal and dermatological (0.87 each). The study determined 3 plant species, i.e., * Acacia modesta *Wall., * Caralluma tuberculata *R.Br., and * Withania somnifera *(L.) Dunal with a FL of 100%. DMR results showed that * Olea ferruginea *(Sol.) Steud. ranked first, * Morus alba *L. ranked second, and * Melia azedarach *L. ranked third. Among the 55 informants, the male concentration was high (61%) and most of them were over 40 years old while a leading quantity of respondents (45%) was uneducated. There is a dire need to take necessary steps for the conservation of important medicinal plants by inhibiting overgrazing and providing alternate fuel resources. Young generations should be educated regarding the importance of ethnomedicinal knowledge and plants with high Fic and FL values should be further checked chemically and pharmacologically for future exploration of modern medicine.

## 1. Introduction

Medicinal plants offer a real substitute in developing countries for the treatment of human and animal ailments [1]. Ethnomedicine is often the single easily reached and affordable therapy available. The world market for herbal medicines based on traditional knowledge is now estimated at US$ 60 billion [2]. Plant-based traditional medicine plays a key role in the development of novelties in drug discovery [3].

Pakistan has a rich medicinal plants history having more than 600 plants being used traditionally for medication [4]. The majority of the medicinal plants are confined to northwest regions of Pakistan due to the presence of Himalayas, Karakoram, Sulaiman, and Hindu Kush mountain ranges [5] that lie in association with Pak-Afghan border. Both countries Pakistan and Afghanistan share almost 2,500 kilometers of boundary, called Durand Line, which was demarcated in 1893 following an agreement between the British Empire and the Afghan king [6]. The Durand Line separates* Pashtun *ethnic group in the Pak-Afghan border areas. Culturally, Pashtuns represent the majority of the populace of Afghanistan and also have significant population in Pakistan. The local language of southeastern Afghanistan and northwestern Pakistan Pashtun ethnic group is* Pashtu* [7]. The majority of the northwest areas of Pakistan living in the proximity to border region are rural in nature with high illiteracy rate and greatly depend on medicinal plants for primary health care and for generating income. In Pakistan, various ethnobotanical studies have been conducted in the different regions [8, 9] (Akhtar et al. [9]; Mussarat et al. [10], Hassan et al. [11]; and Begum et al. [12]). Most of the ethnobotanical studies in Pakistan have documented just the uses of medicinal plants. Almost no studies have been documented on detailed ethnomedicines preparations in the border region villages. The current research is the first effort to provide a thorough overview on the ethnomedicines employed by conventional healers and their detailed appliance in the region. This research will offer baseline data for more comprehensive studies on effectiveness and security of these preparations, as well as the potential applications in the communal health system. Moreover, the region is very rich in medicinal plants due to its conductive climate but this area has never been touched so far. Therefore, it is imperative to document the vegetation and detailed home-grown information of people about medicinal plants of this area before it is lost due to changing cultures. The purpose of this study is to assess traditional medicinal plant knowledge specifically with regard to the traditional healer's demographic characteristics such as gender, age, and source of income and to document the knowledge and the uses of medicinal plants used by the traditional healers in the Hangu region, Pakistan, to provide baseline data for future pharmacological and phytochemical studies by the application of ethnobotanical indices.

## 2. Material and Methods

### 2.1. Study Area

**Figure 1:**
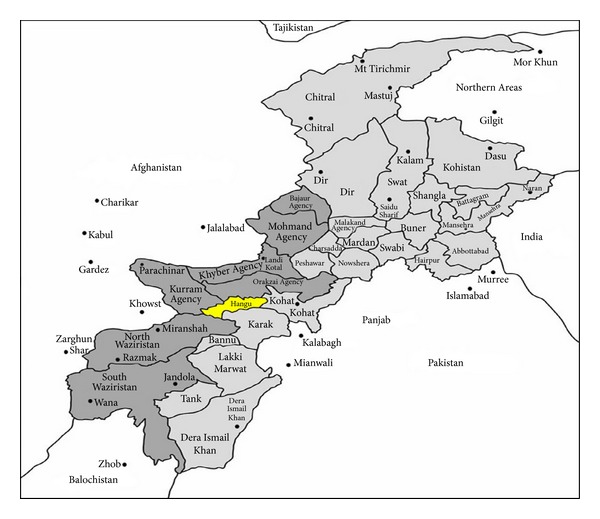
Map of the study area.

The present study was conducted in Hangu district located in north of Khyber Pakhtunkhwa, Pakistan, near the border region with Afghanistan (Figure 1). Hangu is situated at 33.53 North latitude, 71.06 East longitude, and 858 m above the sea level comprising a total area of 1,097 km^2^ and total population of 314,529 [13]. The dominant vegetation in the study area is* Acacia modesta*,* Olea ferruginea, Dodonaea viscosa *(L.) Jacquin,* Acacia nilotica* L.,* Periploca aphylla* Decne.*, Melia azedarach, *and* Morus alba.* The temperature of the area rises gradually from the month of January to June and then slowly turns down up to December. The summer season is modest and warm but June and July are the hottest months. The mean highest temperature is 8.8°C and mean lowest temperature is 7°C in the months of December and January. This district also produces wheat and maize as major crops. The area is rural in nature and the majority of the population is illiterate and they are also deprived of modern health services; hence the locals rely on medicinal flora of the region for the healthcare and to balance their low earnings as well [14]. The inhabitants mostly rely on timber for fuel purposes due to lack of modern fuel resources [13].

### 2.2. Data Collection

Field work was carried out from October 2012 to October 2013. Data was collected by making total eight frequent visits to the study area in four different seasons. Total 250 informants were recommended by knowledgeable elders, local authorities, and development agents. Out of 250, we have randomly selected 55 (34 men and 21 women) out of the total identified key informants. The selected informers were local inhabitants of the area aged between 20 and 89 years. Ethnobotanical survey was taken to gather information on traditional plants utilized by the local healers for the treatment of human ailments in the district following standard methods [15, 16]. The survey was done by using proper semistructured interviews and group discussions as well. A checklist of questions was prepared in English language for undertaking interviews and discussions. The questionnaire contained no strict questions and informants were allowed to speak spontaneously and without pressure. Key questions about medicinal plants were on local name of a particular medicinal plant, types of disease treated, mode and method of remedy preparation, parts of the plants used, use of fresh or dry plant parts, use of single or mixture of plants for remedy preparation, mode of administration, and dose requirement. Sociocultural information about informants was also collected during interview. The informants were interviewed in their local language* Pashto*. Ethically written acceptance was collected from the main office of the district and also from the head person of the village. The purpose of the present study was also explained to each informant in order to remove their hesitation and to encourage them that their knowledge will be a great contribution in the scientific literature.

### 2.3. Specimen Collection and Identification

The reported medicinal plants were collected from natural vegetation and home gardens during the field walks and habits of the plants were listed. The collected voucher specimens were taken to the Herbarium of Kohat University of Science and Technology, Kohat, Pakistan. Specimen identification and confirmation were undertaken by using Flora of Pakistan and taxonomic experts. Specimens with their label were stored at the Herbarium of Kohat University of Science and Technology, Kohat, Pakistan.

### 2.4. Data Organization

The collected ethnobotanical data were entered into Excel spreadsheet 2007 and summarized using graphical statistical methods such as percentages. The habit of the plants was categorized into three categories, that is, herbs, shrubs, and trees. The part used by the healers for the preparation of ethnomedicines was grouped under 11 classes, that is, leaves, whole plant, root, fruit, bark, and so forth. Human ailments treated in the study area were categorized into 18 categories like gastrointestinal, dermatological, skeletomuscular, antidiabetic, and so forth. Route of administration was divided into oral, dermal, and nasal. The basic categorization using questionnaire data regarding informants' sex, age groups, educational status or literacy rate, and occupation was also carried out.

## 3. Data Analyses

### 3.1. Informant Consensus Factor (Fic)

For the analysis of the general use of plants, factor informant consensus (Fic) was used to highlight plants of particular cultural relevance and agreement in the use of plants. Informants' consensus within a community and between cultural groups indicates which plants are widely used and thus aids in the selection of plants for pharmacological and phytochemical studies [17]. In order to use this tool, illnesses were classified into categories, as plants with high Fic are likely to be more pharmacologically efficient as compared to plants with low Fic [18]. Fic values lie between “0.00 and 1.00”. Fic values are always greater when single or few plants are documented to be used by large number of respondents to cure a specific disease, while low Fic values give an indication that informants do not agree over which plant to use [19, 20].

The Fic can be calculated using the formula as follows:

(1) $$\begin{array}{c} \text{Fic}=\frac{\text{nur}-\text{nt}}{\text{nur}-1}, \end{array}$$

where Fic = informants consensus factor, nur = number of use citation in each category, and nt = number of species used.

### 3.2. Fidelity Level (FL)

Fidelity level is useful for identifying the key informants' most preferred species used for treating certain ailments. The medicinal plants that are widely used by the local people have higher FL values than those that are less popular. Fidelity level shows the percentage of informants claiming the use of a certain plant species for the same major purpose. This is designed to quantify the importance of the species for a given purpose. Before calculating the values of FL all of the ailments that were reported are grouped into major classes [21]. FL value was estimated using the formula FL = Ip/Iu × 100, where Ip is the number of respondents who reported the utilization of medicinal plants for a specific main ailment and Iu is the total number of respondents who mentioned the same plant for any ailment [22]. It is assumed that those medicinal plants which are plants that are used in some recurring manner for the same disease category are more likely to be biologically active [18].

### 3.3. Direct Matrix Ranking (DMR)

DMR [15, 16] was used to compare the use diversity of given plant species based on the data collected from the respondents. Total eight informants were selected for the collection of DMR data. Selected informants were asked to assign use values (5 = best, 4 = very good, 3 = good, 2 = less used, 1 = least used, and 0 = not used) to each species. The values (average scores) given to each medicinal plant were summed up and ranked.

## 4. Result and Discussion

### 4.1. Medicinal Plants Reported

The study revealed 67 medicinal plant species belonging to 55 genera and 39 families consisting of 65 angiosperms and 2 gymnosperms in Hangu district (Table 1). The investigated region has a rich diversity of medicinal plants and provides a conductive habitat and ideal climatic conditions for their growth as shown by the presence of 67 medicinal plant species. The majority of the medicinal plants reported were herbs (43%) followed by shrubs (30%) and trees (27%). The high usage of herbs in the study area could be an indication of their abundance and it might also be due to the fact that they are easily accessible near household and might have high effectiveness in the treatment of ailments in comparison to other growth forms [23]. The common use of herbs for medicinal purposes is also reported from other parts of the world [24, 25]. The dominant families in the study area were Asteraceae and Solanaceae represented by the highest number of species (4 species each, 6%) followed by Euphorbiaceae, Moraceae, Oleaceae, and Lamiaceae (3 species each, 4.47%). Other families with low number represented by 2 species each Amaranthaceae, Acanthaceae, Alliaceae, Poaceae, Papilionaceae, Zygophyllaceae, Arecaceae, and Rhamnaceae, while the remaining 22 families had only single species representation. The wider utilization of species from dominant families like Asteraceae and Solanaceae might relate to the presence of effective bioactive ingredients against ailments [26]. Our results are in line with other ethnomedicinal studies conducted in other regions of Pakistan [9, 27] where traditional healers mostly use Solanaceae and Asteraceae members for the preparation of ethnomedicines.

### 4.2. Common Ailments in the Study Area

Traditional healers use 67 medicinal plants for the treatment of a variety of disorders in the studied region. These were grouped into 18 major disease categories like gastrointestinal, dermatological, antipyretic, blood disorders, and so forth. The natives of the region use total 25 plant species for gastrointestinal disorders followed by 13 for dermatological infections. The results are in agreement with other studies conducted in other parts of Pakistan and other countries [28, 29]. The use of a large number of medicinal plants for the treatment of gastrointestinal and dermatological ailments in the region could be due to the high occurrence of these problems in the study area due to bad hygiene, fuel wood smoke inside houses, and other factors like water and air pollution [30]. Informant consensus results have also shown a high degree of consensus for gastrointestinal and dermatological (0.87 each) ailments, which were followed by blood disorder like diabetes (0.84) (Table 2). The highest plant use citation was for gastrointestinal (200) followed by dermatological ailments (100). High Fic value gives an indication that these diseases are more prevalent in the Hangu region that might be due to the poor socioeconomic and sanitary conditions of the people. According to Heinrich et al. [19], high Fic values are very useful in the selection of specific plants for further search of bioactive compounds. The medicinal plants that are widely used by the local people have higher FL values than those that are less popular. The present study revealed 20 medicinal plants having high FL value. FL values in this study varied from 1.0% to 100%. The study determined 3 plant species (*Acacia modesta*,* Caralluma tuberculata,* and* Withania somnifera*) with a FL of 100% followed by 7 (*Allium sativa* L.,* Mentha arvensis* L.,* Mentha longifolia* L.,* Cannabis sativa *L.,* Punica granatum *L.,* Morus alba,* and* Morus nigra* L.) species with more than 90% and less than 100%, which might be taken as a signal of the excellent curative potential of the plants (Table 3). All these plants that reported higher FL values are not only being frequently used in study region but also in other regions of the Pakistan [8, 31]. These plants possess different phytochemicals responsible for their therapeutic actions.* Withania somnifera* contains compound withanolides, which are believed to account for its extraordinary medicinal properties [32].* Caralluma tuberculata* contains pregnane glycosides, flavones glycosides, and other phytochemical responsible for its antidiabetic and anticancer activities [33]. It is understood that plants used in recurring manner are more phytochemically active [16]. High FL value plants might be selected for further chemical screening to investigate the bioactive compounds responsible for their high curative potential [34, 35].

### 4.3. Ethnomedicinal Preparations

**Figure 2:**
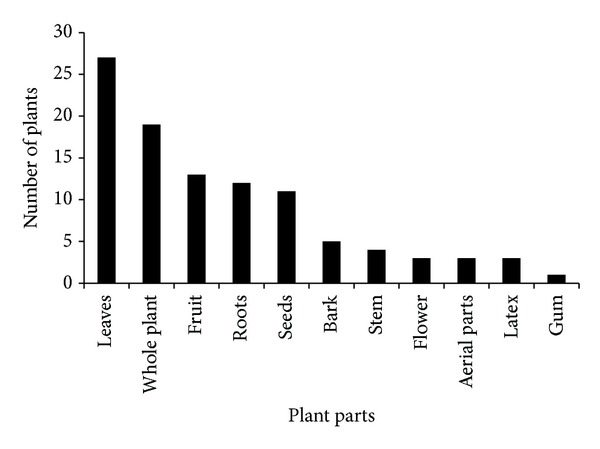
Plant parts used for remedy preparation.

**Figure 3:**
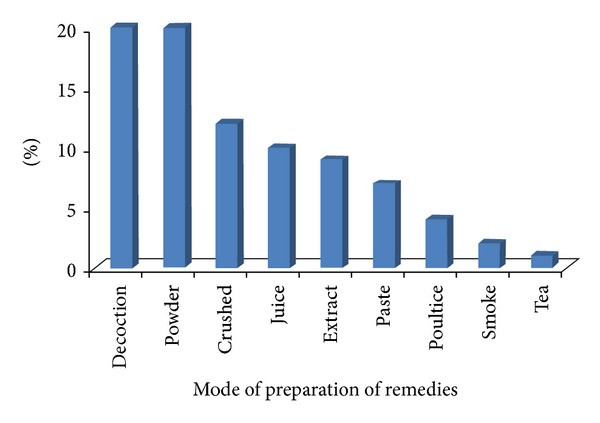
Methods of preparation of ethnomedicines.

Traditional healers mostly use leaves (40%) of the plants followed by whole plant (28%) and fruits (19%) for the preparation of different ethnomedicines. The current investigation showed (Figure 2) that leaves (40%) are the most collected plant parts for medicinal purposes. This might be due to easy availability and containing high amount of chemicals and could be easily extracted and used in different forms but it needs biochemical analysis and pharmaceutical screening to cross-check the local information. Use of leaves of plants does not cause damaging effect on the plant life cycle as compared with other parts like roots and flower, and so forth. Due to good rainfall conditions about eight months in the year, the leaves remain green and abundant for most of the months. Our findings of the frequent use of green leaves in the preparation of remedies corroborate the results of [36, 37]. Traditional healers are involved in preparation of 110 recipes preparation and the major modes of ethnomedicines preparation in the studied region were decoction (20%), powdering (20%), crushing (12%), extracting juice (10%), and so forth (Figure 3). Decoction and grinding of medicinal plants for the preparation of ethnomedicines could be due to their high effectiveness for the curing of various ailments. According to Deeba [38], decoction, grinding or crushing, and boiling methods are the most commonly followed methods for the extraction of active compounds. Monotherapy preparations using single medicinal plant were found to be more abundant in comparison with herbal concoction that was prepared by mixing two or three species; for example, healers take equal amount of extract of onion bulb and mint and mix them for the treatment of cholera. Another example is taking 70 gm dried leaves of wild mint and 30–40 g of “bishops” weed and grinding them together and 10–12 gm of common salt is also added to them and taken for the treatment of gastric problem and stomach pain. The use of mixture of plants has recently been shown to increase the effectiveness of the herbal medicine [39]. Out of total 110 ethnomedicines, 91% were prepared by using fresh plant materials, whereas 9% were prepared using dried parts (Table 1). The high usage of freshly prepared ethnomedicines is an indication of the high abundance of medicinal plants in the surrounding areas to be harvested anytime. These findings are in line with other studies conducted in other areas [40, 41]. The other reason behind the repeated use of fresh plant material could be due to the fact that the drying process contributes to the loss of volatile oil and sometimes due to the fact that high temperature protein becomes denature. Higher use of fresh plant material on the other hand is not a sustainable practice as it may threaten the plants due to recurrent harvesting.

### 4.4. Route of Administration and Dosage

The current survey revealed that most of the plant remedies are taken orally and topically in the investigated region while only single recipe is taken through ear (Table 1). As mentioned earlier, gastrointestinal and skin problems are common in the region and that might be the reason why the majority of the plants are being used orally and topically while some of the plants are being used through ears. Ethnomedicines are taken along different types of additives generally called vectors like honey, salt, sugar, milk, desi ghee, and wheat flour for the purpose of increasing flavor and to reduce the astringent taste of the remedies. This means that since traditional medicines could have sour or bitter tastes in most cases, the additives reduce such tastes and may even improve the efficacy of the medicine. The measurements used to determine the dosages are not standardized and depend on the age and physical appearance of the patient, sociocultural explanation of the illness, diagnosis, and experience of individual herbalist [42]. Mostly the treatment of the patient is completed within a single day or couple of days. When the patients did not show any indication of improvement from their sickness following treatment completion, they were recommended to modern health centres in urban area for further examination by the physician.

### 4.5. Multipurpose Medicinal Plants and Threats to Their Extinction

The results of the DMR implementation on multipurpose medicinal plants enabled us to recognize which of the multipurpose plants are more under stress in the area and the causes that threaten the plant (Table 4). Accordingly,* Olea ferruginea* ranked first,* Morus alba* ranked second,* Melia azedarch* ranked third, and* Acacia modesta* ranked fourth while* Acacia nilotica* ranked fifth. These multipurpose species are basically trees and therefore these species are facing great pressures as the local people are unsustainably harvesting these species for a variety of purposes. The factors responsible for the declining of these species abundance in the area were their overharvesting for agricultural tool, construction, fodder, and fire wood purposes. Beside these major threats, locals of the region also use these plants for handicrafts manufacturing. Free grazing is the common practice in the area. Before the commencement of winter, the grasses are harvested, dried, and put into a stake. The harvesting is done collectively, and then during the bare and cold months of winter, these are fed to the domestic animals. Fuel consumption per home in the studied area is often considered more than the consumption on feeding and other requirements because of severe winters. Thus, the results require urgent conservation strategies to save the declining population of multipurpose plant species in the study region. References [43, 44] have also stated the identical pattern of maximum exploitation of multipurpose medicinal plants for uses other than their traditional medicinal importance in southeastern Ethiopia. Traditional healers mostly use the whole plant of these multipurpose species or individual roots (Table 1) of some species for the preparation of ethnomedicinal recipes and this is an unsustainable practice as compared to leaves. Therefore, there is a dire need to take necessary steps for the conservation of these species before their extinction.

### 4.6. Indigenous Knowledge Associated with Gender, Age, and Socioeconomic Status of the People

Among the 55 informants, 34 (61.81%) were male and 21 (38.18%) were female (Table 5). It is witnessed that males had better knowledge regarding ethnomedicines than females. The reason behind that men have well indigenous knowledge than women might be due to the fact that the men are usually favoured in the shift of the knowledge; however, in many cultures women are responsible for the family's health. The highest number of informants aged above 40 years. The result shows that traditional knowledge is prevalent among the community members; however, it is under threat of transferring to the younger generation to come. The decreasing rate of transfer of indigenous knowledge might be due to the lack of interest among the younger generation to learn and practice it, which might be attributed to the ever increasing influence of modernization [8]. Almost half of the respondents interviewed were illiterate (45.45%), whilst most of those with an education had merely primary (29.09%) which reflect the unavailability of standard educational institution in the area (Table 5). Literate people in the study area were found to have less knowledge of medicinal plants as compared to illiterate ones as the former are more likely to be exposed to modernization as also revealed by studies conducted elsewhere [8, 45, 46]. The inhabitants of the study area are not very well off due to less literacy rate and therefore they are heavily dependent on medicinal plants for a variety of purposes in order to compensate their income.

## 5. Conclusions

The present study has recorded 67 medicinal plants used for the treatment of a variety of human ailments in the rural area near Pak-Afghan border region. In the study area herbs constituted the highest proportion of medicinal plants to be utilized. Mostly the leaves of the plants are harvested for different ethnomedicines preparation. Decoction and powdering are the most common methods of drug preparation and remedies are mostly taken orally in the studied region. A high number of plants have been reported to treat gastrointestinal and dermatological problems. The medicinal plants in the region are also facing some threats like unsustainable collection method of some plants, collection for fuel wood, for construction, and for fodder, and agricultural tools. For sustainable utilization of medicinal plants and to avoid further loss, the District Agricultural Office needs to team up with the local people, by providing the community with planting materials of the most threatened and preferred medicinal and multipurpose species so that they can grow them in their home gardens. Moreover, the documented medicinal plants with high degree of consensus can serve as a basis for future investigation of modern drug.

## Figures and Tables

**Table 1:** Medicinal plants and their preparation methods and administration.

Scientific names	Local names	Families	Habit	Part used	Medicinal uses	Herbal formulation	Administration	Dosage
*Acacia modesta *Wall.	Palosa	Mimosaceae	Tree	Gum, leaves	Backache	Gum and powder of fresh leaves of *Acacia modesta* are mixed with wheat flour and desi ghee and make *Halwa* that is used for backache.	Oral	As needed

*Acacia nilotica *(L.) Delile	Kikar	Mimosaceae	Tree	Whole plant	Narcotic	Extraction of fresh root and leaves are taken as alcohol (*Sharab*).	Oral	As needed
					Aphrodisiac	Four grams of gum is taken as paste with water.	Oral	Once a day
					Earache	About 30 flowers are heated in 10 mL mustard oil and filtered.	Through ear	Two to four drops for 5 days

*Allium cepa *L.	Pyaaz	Alliaceae	Herb	Leaves	Antipyretic	Equal amount of extract of onion bulb and mint are mixed and used against cholera.	Oral	One teaspoon of this mixture is taken per hour for a period as needed.
					Skin infection	Poultice of onion bulb is used against abscesses.	Topical	Two times a day for one week

*Allium sativum *Linn.	Ugga	Alliaceae	Herb	Leaves, roots	Blood disorders	Small pieces of *Allium sativum* are chewed to reduce blood pressure.	Oral	Twice a day
					Gastrointestinal	The powder of leaves and roots are also used against stomach problems.	Oral	As needed

*Aloe barbadensis *Mill.	Zarpati	Aloeaceae	Herb	Leaves	Veterinary (gastrointestinal)	Two leaves are made spineless and each one is divided lengthwise into 2 or 3 slices. These slices of leaves along with common salt are given to the animals for stomach disorders.	Oral	Three doses after every 48 hours period

*Amaranthus viridis* L.	Sarkoomal.	Amaranthaceae	Herb	Leaves	Gastrointestinal	Leaves are crushed with sugar and taken along with black tea for curing constipation.	Oral	Four times a day
					Skin infection	Poultice of leaf is prepared along with mustard oil for the treatment of abscesses.	Topical	As needed

*Anagallis arvensis* L.	Dhabbar	Primulaceae	Herb	Whole plant	Rheumatism	The whole plant is crushed into powder after drying. Two gm of the powder with 5 gm of wheat flour is mixed for the treatment of rheumatism.	Oral	Once a day for a week

*Cannabis sativa* L.	Bhaang	Cannabaceae	Herb	Leaves, flowering tops, and seed	Narcotic	The fruit and leaves are used as narcotic, commonly called “*Charas.*”	Oral	As needed
					Veterinary	The decoction of seeds is given to cattle for increasing milk.	Oral	As needed

*Caralluma tuberculata *R.Br	Pawany	Asclepiadaceae	Shrub	Whole plant	Antidiabetic and anticancer	Whole plant is dried, powdered, and taken with water. Fresh plant is directly eaten by diabetic patient and is very effective in cancer treatment as well.	Oral	Once a day

*Cuscuta reflexa* Roxb.	Chum bud	Cuscutaceae.	Herb	Stem and seeds	Wound	A paste of the plant powder in butter is prepared and is externally applied for wounds.	Topical	As needed
					Skin infection	The whole plant is crushed and then boiled in 8 liters of water for an hour. It is filtered and the patient is advised to take a bath with this decoction without using soap for scabies.	Topical	As needed

*Carthamus oxycantha* Co.Cr.	Spena zagai	Asteraceae	Herb	Seed oil	Jaundice	Seeds are collected, dried under shade, and ground to obtain powder and taken to treat jaundice.	Oral	1 teaspoon of powder is taken twice a day for 3-4 weeks
					Skin infection	Few drops of honey are added in seed powder to make paste. This paste is applied on the face. It is effective to remove white spots of skin.	Topical	As needed

*Citrus sinensis *	Malta	Rutaceae	Shrub	Fruit, leaves	Gastrointestinal	Fruit is eaten for reducing constipation.	Oral	2 fruits per day

*Cynodon dactylon*var. *coursii* (A. Camus) J.R. Harlan and de Wet	Wakha	Poaceae	Herb	Whole plant	Wounds	The paste made of fresh leaves is applied on cuts and bleeding wounds.	Topical	As needed
					Piles	Same as above	Topical	As needed
					Gastrointestinal	Juice of the plant is given in diarrhea.	Oral	Twice a day
					Antipyretic	Same as above	Oral	Twice a day

*Dalbergia sissoo* DC.	Shawa	Papilionaceae	Tree	Whole plant	Piles	70 gm of young leaves of buds are crushed. One glass of water is added to it and strained. The strained decoction is taken daily.	Oral	Taken daily for 10 days
					Jaundice	Same as above	Oral	Taken daily for 10 days

*Datura stramonium* L.	Tora torii.	Solanaceae	Herb	Whole plant	Earache	The juice of flower is useful for earache.	Oral	As needed
					Narcotic	Seeds and leaves are smoked for their narcotic action.	Oral	As needed

*Dicliptera bupleuroides* Nees.	Somni	Acanthaceae	Herb	Whole plant	Skin infection	Poultice is used for scabies.	Topical	Once a day

*Digera muricata* (L.) Mart.	Tandola	Amaranthaceae	Herb	Whole plant	Gastrointestinal	Juice is extracted from the whole plants and used as laxative.	Oral	As needed

*Dodonaea viscosa* (L.) Jacquin	Zetawoni	Sapindaceae	Shrub	Leaves	Rheumatism	The leaves are warmed and kept on joints to relieve pains.	Topical	Once a day

*Eriobotrya japanica* (Thunb.) Lindl.	Lokat	Rosaceae	Tree	Fruit	Chest problems	Fruit is taken directly to treat cough.	Oral	As needed

*Eucalyptus lanceolatus* Dehnh.	Lachi	Myrtaceae	Tree	Whole plant	Gastrointestinal	Leaves and bark are boiled in water. Filtrate and decoction are used for abdominal pains. Fruit is added to green tea and taken as antiemetic.	Oral	Twice a day

*Euphorbia helioscopia* L.	Katta saarai	Euphorbiaceae	Herb	Leaves	Gastrointestinal	Mature leaves (5 g) are mixed with 3 spoonfuls of sugar to prepare recipe to treat constipation	Oral	Twice a day

*Euphorbia hirta* L.	Chapa tray.	Euphorbiaceae	Herb	Whole plant	Diabetes	Leaves juice is taken for diabetes	Oral	As needed

*Fagonia indica* Burm.f.	Mazgha Kai.	Zygophyllaceae.	Herb	Aerial parts	Blood purifier	Extract of aerial parts is used	Oral	Thrice a day
					Skin infection	Same as above	Topical	Thrice a day
					Diabetes	Same as above	Oral	Thrice a day
					Antipyretic	Half kg of the whole plant is boiled in 2 liters of water; patients with hepatitis are advised to take bath with this decoction.	Topical	Thrice a day

*Ficus carica* L.	Inzeer	Moraceae	Tree	Fruit	Piles	Two to four fruits are soaked in water or milk at night and used in the morning on empty stomach.	Oral	Daily for 10 days

*Ficus elastica* Roxb. ex Hornem.	Rubber Plant	Moraceae	Tree	Leaves, Bark	Antipyretic	Leaves and bark are crushed and taken along honey in small quantity to reduce fever.	Oral	Once a day

*Ficus religiosa* L.	Peppal	Moraceae	Tree	Whole plant	Vomiting	Decoction of bark is used.	Oral	As needed

*Foeniculum vulgare* Mill.	Soonphf	Umbelliferae	Herb	Seeds and roots	Gastrointestinal	Take sonf with white zeera, grind it, and use after meal; it is good to remove ulcer and stomach pain	Oral	As needed

*Fumaria indica* (Hausskn.) Pugsley	Khatee soii.	Fumariaceae	Herb	Aerial parts	Blood purification	Two kg of aerial parts is dried under shade and crushed to obtain powder; 2-3 gm powder with one glass of water is taken.	Oral	Twice a day for one week
					Jaundice	Same as above	Oral	Twice a day for one week
					Gastrointestinal	Juice of fresh parts is used as laxative.	Oral	Once a day for four days
					Antipyretic	Juice of fresh parts is used to reduce fever.	Oral	Once a day for two days

*Jasminum humile* f.kensuense	Zeet chumbeli	Oleaceae	Shrub	Flower, root, and latex.	Skin infection	Flowers and roots are boiled to make paste and rub on skin for treating pimples.	Topical	Twice a day for one week

*Jasminum officinale* L.	Chumbeli	Oleaceae	Shrub	Whole plant.	Gastrointestinal	Decoction of leaves and roots are prepared and used as anthelmintic.	Oral	Once a day
					Kidney problems	Crushed leaves are mixed with flour and taken along water to treat kidney stones.	Oral	Twice a day for one month

*Justicia adhatoda* L	Shna Baza	Acanthaceae	Shrub	Leaves	Diabetes	Half kg of fresh leaves of this plant is extracted with 500 mL water and used against diabetes.	Oral	10 mL of extract is used twice a day
					Blood purification	Same as above	Oral	10 mL of extract is used twice a day
					Chest infection	Leaves and flowers are plucked, dried under shade, ground to obtain powder; 50 gm of this powder is mixed in 15 mL of honey.	Oral	Half teaspoon twice a day for 15 days
					Skin infection	Half kg leaves are boiled in 4 liters of water and decoction is used.	Oral	Twice a day

*Lathyrus aphaca* L.	Jee Wareen	Papilionaceae	Herb	Seed and flower	Skin infection	Decoction is used for skin problems.	Topical	As needed

*Malva neglecta* Wallr.	Panderak	Malvaceae	Herb	Whole plant	Kidney problems	Roots are taken and boiled in 2 glasses of water and after boiling when 1 glass of water remains, it is taken for kidney stones.	Oral	Once a day for 40 days

*Melia azedarach* L.	Tora Draka	Meliaceae	Tree	Whole plant	Diabetes	Powder of seeds is used.	Oral	As needed
					Gastrointestinal	Fruit is ground and its juice is mixed with oil and taken as anthelmintic.	Oral	As needed

*Mentha arvensis* L.	Podeena.	Lamiaceae.	Herb	Leaves	Gastrointestinal	70 gm dried leaves of wild mint and 30–40 gm of bishops' weed are ground together and 10–12 gm of common salt is also added. It is used for gas problems and stomach pain.	Oral	Thrice a day after meal
					Vomiting	Tea of dried leaves is taken to stop vomiting.	Oral	As needed

*Mentha longifolia* L.	Venalai	Lamiaceae	Herb	Leaves	Gastrointestinal	Decoction of leaves is used as carminative.	Oral	As needed

*Monotheca buxifolia* (Falc.) A. DC.	Gorgola	Sapotaceae	Shrub	Fruit, stem	Skin infection	Poultice is used against skin infection.	Topical	As needed

*Morus alba* L.	Toot	Moraceae	Tree	Fruit, leaves	Gastrointestinal	Crushed leaves are taken along honey to treat diarrhea.	Oral	Twice a day

*Morus nigra* L.	Tor Toot	Moraceae	Tree	Fruit, leaves	Kidney problems	Fruit is directly eaten as diuretic.	Oral	Twice a day

*Nannorrhops ritchiana*. (Griff.) Aitch.	Mazzari	Arecaceae/Palmae	Shrub	Leaves	Gastrointestinal	Crushed leaves are used as carminative.	Oral	As needed
					Veterinary	Fresh leaves are given to animals as purgative.	Oral	As needed

*Nerium oleander* L.	Gand derai	Apocynaceae	Shrub	Leaves	Dental pain Wound	The fresh leaves are washed and crushed, and then 3 cups of water are added. The filtrate is given to the patients suffering from dental pain.	Oral	Twice a day for 5 days
						Poultice of leaves is applied externally to reduce swelling.	Topical	Twice a day

*Olea ferruginea* (Sol.) Steud.	Kawwaan	Oleaceae.	Tree	Fruit, leaves, seeds, and bark	Dental	Decoction is used for toothache.	Oral	The decoction of fresh leaves is kept in the mouth at night till recovery.
					Rheumatism	The oil extracted from the fruits is used as massage in the treatment of rheumatism.	Topical	As needed
					Skeletomuscular	Same as above	Topical	As needed

*Otostegia limbata* Benth.	Spin azghai	Lamiaceae	Shrub	Whole plant	Throat infection	50 gm fresh leaves are ground and 3-4 teaspoons of water are added to it. This mixture is filtered through a cloth and is given to the patient suffering from mouth gums and throat pains.	Oral	As needed
					Wound	Crushed leaves are applied for curing of wounds.	Topical	Once a day

*Oxalis corniculata* L.	Tokee pi.	Oxalidaceae	Herb	Leaves, root	Gastrointestinal	Juice of leaves and roots are used against stomach problem.	Oral	As needed

*Peganum harmala *L.	Spin nali	Zygophyllaceae	Herb	Seeds	Spiritual	The smoke from burning seeds and leaves is believed to be devil repellent and also used as protection against evil eyes.		

*Periploca aphylla* Decne.	Barada	Periplocaceae	Shrub	Stem, bark, and latex.	Gastrointestinal	Branches and flower are dried under shade, ground to obtain powder, and taken along water for constipation and stomach ulcer.	Oral	2–4 gm of is powder twice a day

*Phoenix dactylifera* L.	Khajoor	Arecaceae	Tree	Fruit, leaves	Gastrointestinal	Take four dried khobani and three khajoor and keep it in milk and boil it. After cooling take it on an empty stomach with 1 teaspoon of isapagul; it is good for controlling constipation.	Oral	As needed
					Sex power	Same as above	Oral	As needed

*Pinus roxburghii* Sarg.	Nakthar	Pinaceae	Tree	All aerial	Dental	Juice is extracted from fresh leaves and bark by grinding. This is mixed with water and taken for toothache.	Oral	Twice a day before meal and at bed time
					Gastrointestinal	Similarly, the bark and leaves of *Pinus* are dried and crushed, and then the powder is dissolved in cold water and taken for diarrhea.	Oral	Twice a day before meal and at bed time
					Skin infection	Leaves of the plant are boiled and the extract is obtained and taken before meal as remedy for scabies.	Oral	As needed

*Pistacia chinensis* Bunge.	Shenai		Tree	Whole plant	Gastrointestinal	Powdered galls fried with ghee are given internally in dysentery.	Oral	Once a day
					Skin infections	The stem gum is added to the mustard oil, warmed, and mixed. The prepared poultice is then applied to the ruptured heels at night.	Topical	At night for once

*Plantago lanceolata* L.	Gwayo zhabe	Plantaginaceae	Herb	Whole plant	Dental	Leaves are crushed and kept in mouth to relieve toothache.	Oral	As needed
					Gastrointestinal	Seeds and fruits are drunk as purgative and laxative.	Oral	As needed
					Skin infections	Fresh leaves are crushed for athlete's foot.	Topical	As needed

*Plantanus orientalis* L.	Chenar	Platanaceae	Tree	Whole plant	Gastrointestinal	The peel of the fruit is dried, soaked, and ground. The powder so formed, called “Narsaway,” is mixed in small quantity in a cup of curd and is used for dysentery.	Oral	Twice in a day till recovery for the treatment

*Punica granatum* L.	Anar	Punicaceae	Shrub	Fruit, bark	Gastrointestinal	The fruit pericarp is dried, powdered, mixed with sugar, and used for diarrhea and dysentery.	Oral	As needed
					Chest infections	The fruit pericarp is mixed with tea and is given for whooping cough.	Oral	As needed
					Blood purifier	Fruit is directly eaten.	Oral	As needed

*Rumex dentatus* L.	Reen zakai	Polygonaceae	Herb	Leaves	Sex enhancer	Decoction of leaves is used.	Oral	Once at night
					Skeletomuscular	Same as above	Oral	As needed

*Ricinus communis* L.	Raanda	Euphorbiaceae	Shrub	Seeds, leaf, bark, and root.	Gastrointestinal	The small quantity of oil is rubbed on the abdomen, which is slowly and gradually absorbed through sweat glands to release constipation.	Topical	Twice a day for one day

*Sageretia thea* (Osbeck) M.C. Johnst.	Mamoti	Rhamnaceae	Shrub	Fruit, roots	Jaundice	The extraction of roots is used as cooling agent in jaundice.	Oral	Once a day

*Saccharum spontaneum* L.	Shaat	Poaceae	Herb	Whole plant	Chest infection	Juice of whole plants is mixed with milk for the treatment of cough.	Oral	Twice a day for two days

*Solanum incanum* L.	Tarkha Mowtngee	Solanaceae	Shrub	Leaves and roots	Kidney problems	Decoction of leaves and roots are used to break kidney stones.	Oral	As needed

*Solanum villosum* Miller.	Koot soab	Solanaceae	Herb	Whole plant	Kidney problems	Decoction of leaves and roots are used to break kidney stones.	Oral	As needed

*Sonchus arvensis* L.	Kroo Konai	Asteraceae	Herb	Whole plant	Wounds	The whole plant is crushed to form a paste. The paste is applied as a poultice on wounds and boils.	Topical	As needed

*Silybummarianum* (L.)	Azghai	Asteraceae	Herb	Leaves, seeds, and flowers head	Antipyretic Jaundice Liver problems	Seeds are collected, dried under shade, and roasted in vegetable oil. Roasted seeds are ground to obtain powder. This is used to treat hepatitis.	Oral	Half teaspoon of this powder is taken thrice a day for a month
						Same as above	Oral	Same as above
						Same as above	Oral	Same as above

*Taraxacum officinale* F.H. Wigg.		Asteraceae	Herb	Leaves, root	Jaundice	20–30 gm dried aerial parts are boiled in 1 liter of water for 15–20 min to which 15–20 gm sugar is added. This decoction is filtered and used against jaundice.	Oral	Half cup is given twice a day
					Diabetes	Half kg dried aerial parts are boiled in 2 liters water and decoction is filtered and used for diabetes.	Oral	One cup of this decoction is taken twice a day

*Terminalia arjuna* L.		Combretaceae	Tree	Bark, fruits, and leaves	Cardiovascular	Fruits and leaves are ground to make powder and mix with essential additives.	Oral	Once a day

*Vitex negundo* L.	Marmandi	Verbenaceae	Shrub	Leaves, root, stem, and seeds	Gastrointestinal	60 gm dried seeds of this plant, 30 gm Bishop's weed, and 2-3 teaspoon of common salt are ground together to powder.	Oral	As needed
					Antipyretic	Same as above	Oral	As needed
					Jaundice	The decoction of leaves is used for jaundice.	Oral	As needed
					Kidney problem	The seeds are ground to obtain powder and are taken with water for kidney stone.	Oral	Half spoon once a day

*Withania somnifera* (L.) Dunal	Kapyanga	Solanaceae	Herb	Leaves, roots, and seeds	Kidney problems	The decoction of leaves is taken to break kidney stones.	Oral	As needed

*Ziziphus mauritiana* var.* abyssinica *(Hochst. ex A. Rich.) Fiori	Bera	Rhamnaceae	Tree	Fruit, root, and leaves	Gastrointestinal	The decoction of fruit and bark is taken with a cup of milk to treat constipation and dysentery.	Oral	As needed

*Ziziphus nummularia* (Burm. f.) Wight and Arn.	Karkata	*Rhamnaceae *	Shrub	Fruit, leaves	Gastrointestinal	Powder of fruits and leaves are used to treat constipation.	Oral	Thrice a day for 2 days

**Table 2:** Fic values of traditional medicinal plants for treating human ailments in district Hangu.

S. Number	Disease categories	Nur	Nt	Fic
1	Gastrointestinal	200	25	0.87
2	Dermatological	100	13	0.87
3	Skeletomuscular	7	2	0.83
4	Blood disorders	58	10	0.84
5	Chest infections	24	7	0.73
6	Jaundice	14	7	0.53
7	Ear nose throat problems	12	3	0.81
8	Antipyretic	32	7	0.80
9	Narcotic	3	2	0.54
10	Sex power	8	3	0.71
11	Kidney problems	22	7	0.71
12	Wounds	3	2	0.54
13	Rheumatism	9	3	0.75
14	Veterinary	9	3	0.75
15	Dental	12	4	0.71
16	Piles	9	3	0.75
17	Liver problems	7	1	1
18	Cardiovascular	9	1	1

**Table 3:** Fidelity level value of medicinal plants commonly reported against a given ailment.

Number	Medicinal plants	Ailments	lp	lu	FL value %
01	*Acacia modesta *	Skeletomuscular	19	19	100
02	*Caralluma tuberculata *	Antidiabetic	19	19	100
03	*Withania somnifera *	Gastrointestinal	26	26	100
04	*Allium sativum *	Blood pressure	18	19	94.7
05	*Mentha arvensis *	Gastrointestinal	23	25	92
06	*Mentha longifolia *	Gastrointestinal	23	25	92
07	*Cannabis sativa *	Narcotic	11	12	91.6
08	*Punica granatum *	Blood purifier	21	23	91.3
09	*Morus alba *	Respiratory tract	19	21	90.4
10	*Morus nigra *	Respiratory tract	19	21	90.4
11	*Oxalis corniculata *	Gastrointestinal	17	19	89.4
12	*Fagonia indica *	Dermatological	17	19	89.4
13	*Fagonia indica *	Blood purifier	26	30	86.6
14	*Ricinus communis *	Pregnancy	06	08	75
15	*Olea ferruginea *	Dermatological	12	16	75
16	*Olea ferruginea *	Sore throat	11	15	73.3
17	*Justicia adhatoda *	Skeletomuscular	07	10	70
18	*Cuscuta reflexa *	Dermatological	11	16	68.7
19	*Ziziphus nummularia *	Antidiabetic	06	10	60
20	*Sageretia thea *	Antidiabetic	08	14	57.1

**Table 4:** DMR score of fifteen key informants for eleven medicinal plants species with additional uses besides medicinal value.

Use diversity	*A. modesta *	*P. chinensis *	*D. viscosa *	*D. sissoo *	*M. azedarach *	*M. alba nigra *	*O. ferruginea *	*F. religiosa *	*P. roxburghii *	*A. nilotica *	*Z. mauritiana *	Total	Rank
Agricultural tool	2	0	3	0	4	5	5	0	1	5	0	**25**	**4**
Construction	0	4	0	5	5	5	5	5	4	3	2	**38**	**3**
Fodder	5	3	0	0	0	5	3	3	0	0	4	**23**	**5**
Fire wood	5	3	5	3	5	4	5	4	3	5	3	**45**	**1**
Medicine	5	3	3	3	4	5	5	2	3	3	3	**39**	**2**
Total	**17**	**13**	**11**	**11**	**18**	**20**	**23**	**14**	**11**	**16**	**12**		
Rank	**4**	**7**	**9**	**9**	**3**	**2**	**1**	**6**	**9**	**5**	**8**		

Based on use criteria (5 = best; 4 = very good; 3 = good; 2 = less used; 1 = least used; and 0 = no value).

**Table 5:** Gender, age group and literacy level frequencies, and occupation of the interviewed people in the region.

	Total	Percentage
Gender		
Male	34	61.81
Female	21	38.18
Age groups		
20–29	2	3.63
30–39	3	5.45
40–49	7	12.72
50–59	7	12.72
60–69	15	27.27
70–79	12	21.81
80–89	9	16.3
Educational attainment		
Illiterate	25	45.45
Primary	16	29.09
Middle	10	18.18
Secondary	2	3.63
University	2	3.63
Occupation		
Females		
House wives	19	90.47
Primary teacher	2	9.52
Males		
Shopkeepers	10	29.4
Farmers	13	38.2
Labours	6	17.6
Primary teachers	5	14.7

## References

[1] Feo DV (1992). Medicinal and magical plants in the northern Peruvian Andes. *Fitoterapia*.

[2] Breevort P (1998). The booming U.S. botanical market: a new overview. *Herbal Gram*.

[3] Wright CW (2005). Plant derived antimalarial agents: new leads and challenges. *Phytochemistry Reviews*.

[4] Hamayun M, Khan SA, Kim HY, Leechae IJ (2006). Traditional knowledge and ex-situ conservation of some threatened medicinal plants of Swat Kohistan. *Pakistan Journal of Botany*.

[5] Hamayun S (2011). Structural diversity, vegetation dynamics and anthropogenic impaction Lesser Himalayan subtropical Forests of Bagh District, Kashmir. *Pakistan Journal of Botany*.

[6] Mohammad S (2010). *Pakistan-Afghanistan: The Conjoined Twins*.

[7] Barnett RR, Abubakar S (2006). *Resolving Pakistan-Afghanistan Stalemate*.

[8] Adnan M, Ullah I, Tariq A (2014). Ethnomedicine use in the war affected region of northwest Pakistan. *Journal of Ethnobiology and Ethnomedicine*.

[9] Akhtar N, Rashid A, Murad B, Bergmeier E (2013). Diversity and use of ethnomedicinal plants in the region of Swat, North Pakistan. *Journal of Ethnobiology and Ethnomedicine*.

[10] Mussarat S, AbdElsalam NM, Tariq A, Wazir SM, Ullah R, Adnan M (2014). Use of ethnomedicinal plants by the people living around Indus River. *Evidence-Based Complementary and Alternative Medicine*.

[11] Hassan IH, Murad W, Tariq A, Ahmad A (2014). Ethnoveterinary study of medicinal plants in Malakand Valley, District Dir (Lower), Khyber Pakhtunkhwa, Pakistan. *Irish Veterinary Journal*.

[12] Begum S, AbdElsalam NM, Adnan M, Tariq A, Yasmin A, Hameed R (2014). Ethnomedicines of highly utilized plants in temperate Himalaya region. *African Journal of Traditional Complementary and Alternative Medicine*.

[13] Khan I (2013). *Ethnobotanical and ecological study of Hangu district, Pakistan [M.S. thesis]*.

[14] Mustafa G (2009). Education policy analysis report of Khyber Pakhtunkhwa.

[15] Martin GJ (1995). *Ethnobotany: A Method Manual*.

[16] Cotton CM (1996). *Ethnobotany: Principles and Applications*.

[17] Giday M, Teklehaymanot T, Animut A, Mekonnen Y (2007). Medicinal plants of the Shinasha, Agew-awi and Amhara peoples in northwest Ethiopia. *Journal of Ethnopharmacology*.

[18] Trotter RT, Logan MH, Etkin NL (1986). Informants consensus: a new approach for identifying potentially effective medicinal plants. *Plants in Indigenous Medicine and Diet*.

[19] Heinrich M, Ankli A, Frei B, Weimann C, Sticher O (1998). Medicinal plants in Mexico: healers’ consensus and cultural importance. *Social Science and Medicine*.

[20] Canales M, Hernandez T, Caballero J (2005). Informant consensus factor and antibacterial activity of the medicinal plants used by the people of San Rafael Coxcatlán, Puebla, México. *Journal of Ethnopharmacology*.

[21] Giday M, Asfaw Z, Woldu Z, Teklehaymanot T (2009). Medicinal plant knowledge of the Bench ethnic group of Ethiopia: an ethnobotanical investigation. *Journal of Ethnobiology and Ethnomedicine*.

[22] Friedman J, Yaniv Z, Dafni A, Palewitch D (1986). A preliminary classification of the healing potential of medicinal plants, based on a rational analysis of an ethnopharmacological field survey among Bedouins in the Negev Desert, Israel. *Journal of Ethnopharmacology*.

[23] Singh GA, Kumar A, Tewari DD (2012). An ethnobotanical survey of medicinal plants used in Terai forest of western Nepal. *Journal of Ethnobiology and Ethnomedicine*.

[24] Tabuti JRS, Lye KA, Dhillion SS (2003). Traditional herbal drugs of Bulamogi, Uganda: plants, use and administration. *Journal of Ethnopharmacology*.

[25] Uniyal SK, Singh KN, Jamwal P, Lal B (2006). Traditional use of medicinal plants among the tribal communities of Chhota Bhangal, Western Himalaya. *Journal of Ethnobiology and Ethnomedicine*.

[26] Gazzaneo LRS, Lucena RFP, Albuquerque UP (2005). Knowledge and use of medicinal plants by local specialists in an region of Atlantic Forest in the state of Pernambuco (Northeastern Brazil). *Journal of Ethnobiology and Ethnomedicine*.

[27] Adnan M, Begum S, Latif A, Tareen AM, Lee LJ (2012). Medicinal plants and their uses in selected temperate zones of Pakistani Hindukush-Himalaya. *Journal of Medicinal Plants Research*.

[28] Murad W, Ahmad A, Gilani SA, Khan MA (2011). Indigenous knowledge and folk use of medicinal plants by the tribal communities of Hazar Nao Forest, Malakand District, North Pakistan. *Journal of Medicinal Plant Research*.

[29] Tolossa K, Debela E, Athanasiadou S, Tolera A, Ganga G (2013). Ethno-medicinal study of plants used for treatment of human and livestock ailments by traditional healers in South Omo, Southern Ethiopia. *Journal of Ethnobiology and Ethnomedicine*.

[30] Azizullah A, Khattak MNK, Richter P, Häder D-P (2011). Water pollution in Pakistan and its impact on public health—a review. *Environment International*.

[31] Alamgeer, Ahmad T, Rashid M (2013). Ethnomedicinal Survey of plants of Valley Alladand Dehri, Tehsil Batkhela, District Malakand, Pakistan. *International Journal of Basic Medical Sciences and Pharmacy*.

[32] Bhattacharya SK, Bhattacharya D, Sairam K, Ghosal S (2002). Effect of *Withania somnifera* glycowithanolides on a rat model of tardive dyskinesia. *Phytomedicine*.

[33] Rauf A, Jan MR, Rehman WU, Muhammad N (2013). Phytochemical, phytotoxic and antioxidant profile of *Caralluma tuberculata* N. E. Brown. *Wudpecker Journal of Pharmacy and Pharmacology*.

[34] Bekalo TH, Woodmatas SD, Woldemariam ZA (2009). An ethnobotanical study of medicinal plants used by local people in the lowlands of Konta Special Woreda, southern nations, nationalities and peoples regional state, Ethiopia. *Journal of Ethnobiology and Ethnomedicine*.

[35] Lulekal E, Asfaw Z, Kelbessa E, Damme VP (2013). Ethnomedicinal study of plants used for human ailments in Ankober District, North Shewa Zone, Amhara Region, Ethiopia. *Journal of Ethnobiology and Ethnomedicine*.

[36] Kala CP (2005). Ethnomedicinal botany of the Apatani in the Eastern Himalayan region of India. *Journal of Ethnobiology and Ethnomedicine*.

[37] Bhat JA, Kumar M, Bussmann RW (2013). Ecological status and traditional knowledge of medicinal plants in Kedarnath Wildlife Sanctuary of Garhwal Himalaya India. *Journal of Ethnobiology Ethnomedicine*.

[38] Deeba F (2009). *Documentation of ethnoveterinary practices in urban and peri-urban areas of Faisalabad, Pakistan [Ph.D. thesis]*.

[39] Zonyane S, van Vuuren SF, Makunga NP Pharmacological and phyto-chemical analysis of a medicinal plant mixture that is used as traditional medicine in Western Cape.

[40] Ignacimuthu S, Ayyanar M, Sivaraman K (2006). Ethnobotanical investigations among Tribes in Madurai District of Tamil Nadu (India). *Journal of Ethnobiology and Ethnomedicine*.

[41] Bussmann RW, Sharon D (2006). Traditional medicinal plant use in Loja Province, Southern Ecuador. *Journal of Ethnobiology and Ethnomedicine*.

[42] Jabbar A, Raza MA, Iqbal Z, Khan MN (2006). An inventory of the ethnobotanicals used as anthelmintics in the southern Punjab (Pakistan). *Journal of Ethnopharmacology*.

[43] Sher Z, Khan DZ, Hussain F (2011). Ethnobotanical studies of some plants of Chagharzai Valley, District Buner, Pakistan. *Pakistan Journal of Botany*.

[44] Yineger H, Kelbessa E, Bekele T, Lulekal E (2007). Ethnoveterinary medicinal plants at Bale Mountains National Park, Ethiopia. *Journal of Ethnopharmacology*.

[45] Gedif T, Hahn H (2003). The use of medicinal plants in self-care in rural central Ethiopia. *Journal of Ethnopharmacology*.

[46] Bastien JW (1982). Exchange between Andean and Western medicine. *Social Science and Medicine*.

